# Supplementary material for: Synthesis and Biological Assessment of 4,1-Benzothiazepines with Neuroprotective Activity on the Ca2+ Overload for the Treatment of Neurodegenerative Diseases and Stroke
Source: Molecules. 2021 Jul 24;26(15):4473. doi: 10.3390/molecules26154473 (PMC8347512; doi:10.3390/molecules26154473)
Supplement: Supplementary file 1 [file molecules-26-04473-s001.zip › molecules-1274495-SI.pdf]

Supporting Info

Synthesis and biological assessment of 4,1-benzothiazepines with neuroprotective activity on the Ca^{2+} overload for the treatment of neurodegenerative diseases and stroke

Lucía Viejo[1,2], Marcos Rubio-Alarcón[1], Raquel L. Arribas[1], Manuel Moreno-Castro[1], Raquel Pérez-Marín[1], María Braun-Cornejo[1], Martín Estrada-Valencia[1,3],* Cristóbal de los Ríos[1,2,]*

[1]Instituto Teófilo Hernando and Departmento de Farmacología y Terapéutica, Universidad Autónoma de Madrid, C/ Arzobispo Morcillo, 4, 28029, Madrid, Spain; [2]Instituto de Investigación Sanitaria, Servicio de Farmacología Clínica, Hospital Universitario de la Princesa, 28006 Madrid, Spain.
[3]Present address: Leeds Institute of Cardiovascular and Metabolic Medicine. School of Medicine. University of Leeds

*Corresponding authors: Martín Estrada-Valencia, Cristóbal de los Ríos, e-mail addresses: cristobal.delosrios@inv.uam.es; M.H.Estrada-Valencia@leeds.ac.uk

Contents

Synthesis of precursors and intermediates S2

^{1}H and ^{13}C-NMR spectra of final compounds S6

Table of prediction of Lipinski's parameters S18

References S19

General procedure for the synthesis of isopropylbenzaldehydes

Similar to what has been described, (Anajota, 2015) with slight modifications. To a solution of the corresponding bromoisopropylbenzene (1 equiv) in dry THF (15 mL) at –78 ºC under argon, *n*-butyllithium (2.5 M in hexanes, 1.1 equiv) was added dropwise and the resulting mixture was stirred at –78 ºC for 30 min. Then, *N,N*-dimethylformamide (1.1 equiv) was added and the mixture stirred at –78 ºC for 30 min more, then allowed to warm it up –20 ºC and stirred until the disappearance of reagents monitored by TLC (2–5 h). Reaction was stopped with H_2O (30 mL) and extracted with diethyl ether (3 × 50 mL). The combined organic layer was dried over anhydrous $MgSO_4$, filtered, and the solvent removed by vacuum obtaining a colorless oil that did not need further purification.

- p-*isopropylbenzaldehyde*

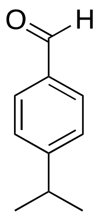

Following the *General procedure for the synthesis of isopropylbenzaldehydes*, reaction of 1-bromo-4-isopropylbenzene (2.51 mmol, 500 mg) [*n*-BuLi (2.76 mmol) and *N,N*-dimethylformamide (2.76 mmol, 202 mg, 214 μL)] yielded *p*-isopropylbenzaldehyde as a yellow oil (372 mg, quantitative yield) with spectral data according to literature [1].

- meta-*isopropylbenzaldehyde*

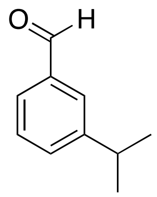

Following the *General procedure for the synthesis of isopropylbenzaldehydes*, reaction of 1-bromo-3-isopropylbenzene (3.07 mmol, 611 mg) [*n*-BuLi (3.38 mmol) and *N,N*-dimethylformamide (3.38 mmol, 247 mg, 261 μL)] yielded *m*-isopropylbenzaldehyde as a yellow oil (453 mg, quantitative yield) with spectral data according to literature [1].

Synthesis of 4-dimethylamino-*N*-tert-butoxycarbonylaniline (19).

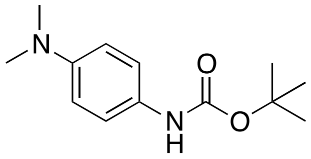

Similar to what we have described for other Boc-protected anilines, (Anajota, 2015) with slight modifications. *N,N*-dimethyl-p-phenylenediamine (1 g, 7.34 mmol) and tert-butyl dicarbonate (1.76 g, 8.08 mmol) were dissolved in dry tetrahydrofurane (THF) under Ar, and triethylamine (810 mg, 1.12 mL, 8.08 mmol) was injected slowly. The reaction mixture was stirred at room temperature for 2 h, after then solvent was evaporated. The crude was dissolved in ethyl acetate (30 mL) and washed with saturated $NaHCO_3$ (3 × 10 mL) and brine (3 × 10 mL). The combined organic layer was dried over Na_2SO_4, filtered and evaporated, obtaining a solid in quantitative yield with spectral data according to the literature [2].

Synthesis of *N*-[4-(dimethylamino)phenyl]-2,2-dimethylpropanamide (22).

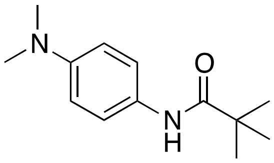

N,N-dimethyl-p-phenylenediamine (1 g, 7.34 mmol) was dissolved in dry CH_2Cl_2 (11 mL), and triethylamine (1.28 mL, 928 mg, 9.175 mmol) was slowly added under Ar. The mixture was cooled down to 0 ºC and pivaloyl chloride (0.99 mL, 973 mg, 8.07 mmol) was injected dropwise, allowing the reaction to stir at 0 ºC for 15 min. Then, the reaction mixture was warmed up to rt and stirred for 3 h, monitoring reaction evolution by TLC. Reaction was terminated by addition of water (10 mL) and washed with saturated $NaHCO_3$ (3 × 10 mL). Combined organic layer was dried using anhydrous Na_2SO_4, filtered, and the solvent evaporated under vacuum. Compound **22** (1.53 g, 95%) was obtained as an intense purple-colored solid. ^{1}H NMR (300 MHz, $CDCl_3$) δ 7.35 (d, J = 9.0 Hz, 2H, H2, H6), 6.69 (d, J = 9.0 Hz, 2H, H3, H5), 7.20 (bs, 1H, NH), 2.90 [s, 6H, $N(CH_3)_2$], 1.29 [s, 9H, $C(CH_3)_3$].

Synthesis of *N*-phenyl-2,2-dimethylpropanamide (29).

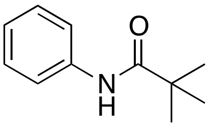

Aniline (511 mg, 5.49 mmol) was dissolved in dry CH_2Cl_2 (9 mL), and triethylamine (0.96 mL, 694 mg, 6.86 mmol) was slowly added under Ar. The mixture was cooled down to 0 ºC and pivaloyl chloride (0.67 mL, 662 mg, 5.49 mmol) was injected dropwise, allowing the reaction to stir at 0 ºC for 15 min. Then, the reaction mixture was warmed up to rt and stirred for 3 h, monitoring reaction evolution by TLC. Reaction was terminated by addition of water (10 mL) and washed with saturated $NaHCO_3$ (3 × 10 mL). Combined organic layer was dried using anhydrous Na_2SO_4,

filtered, and the solvent evaporated under vacuum. Compound **29** (974 mg, > 99%) was obtained as a yellow solid, possessing spectral data according with the literature [3].

Synthesis of 2,2-dimethyl-*N*-(4-pyridinyl)propanamide (34).

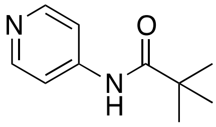

Similarly to what has been described [4] with slight modifications, 4-aminopyridine (2 g, 20.42 mmol) was dissolved in dry CH_2Cl_2 (30 mL), and triethylamine (3.6 mL, 2.6 g, 25.69 mmol) was slowly added under Ar. The mixture was cooled down to –20 ºC and pivaloyl chloride (2.84 mL, 2.78 g, 23.06 mmol) was injected dropwise, allowing the reaction to stir at –20 ºC for 75 min, monitoring reaction evolution by TLC. Reaction was terminated by addition of water (30 mL) and washed with saturated $NaHCO_3$ (3 × 50 mL). Combined organic layer was dried using anhydrous Na_2SO_4, filtered, and the solvent evaporated under vacuum. The crude was purified by automatized flash chromatography (Biotage Isolare One) using ethyl acetate/*n*-hexane mixtures as eluent to give compound **34** (2.2 g, 65%) was obtained as a white solid, possessing spectral data according with the literature [4].

Spectral data

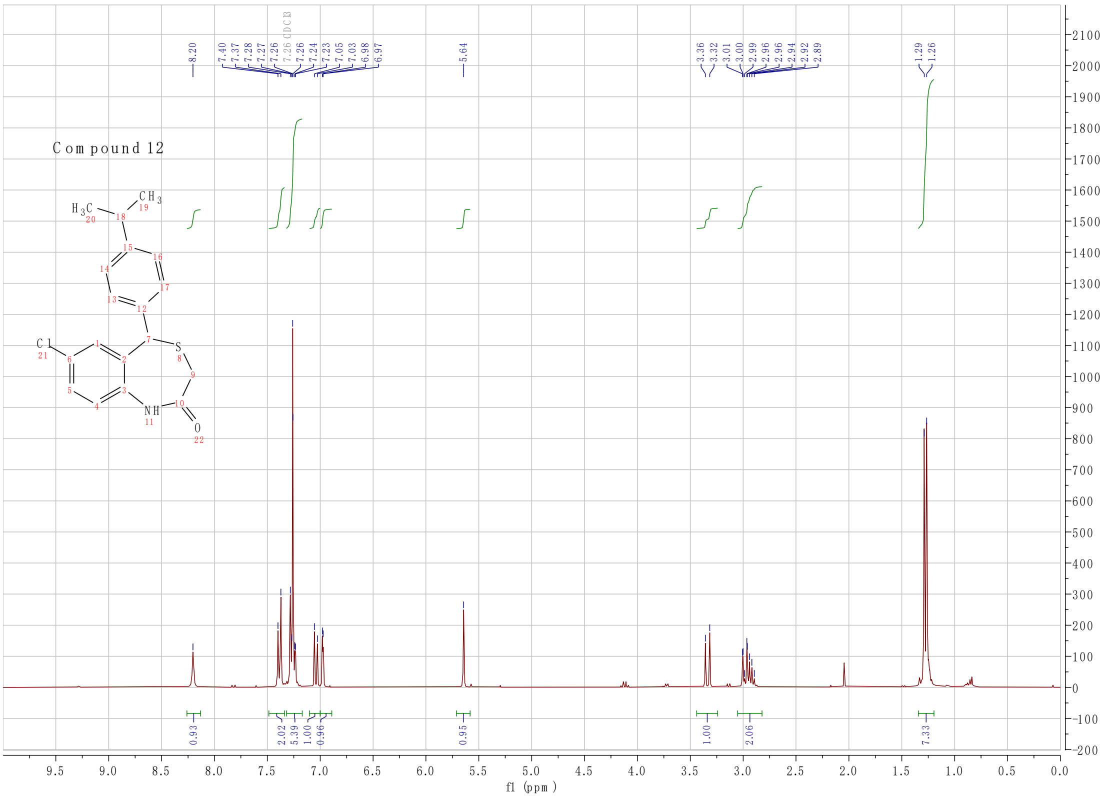

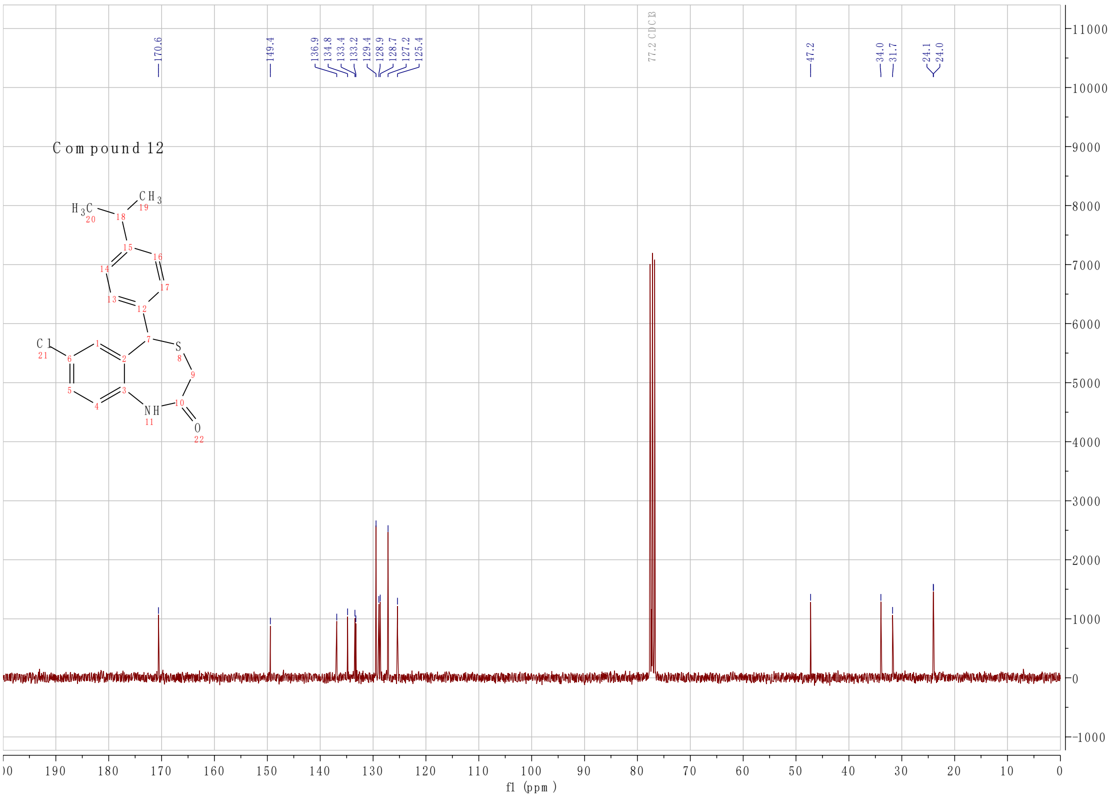

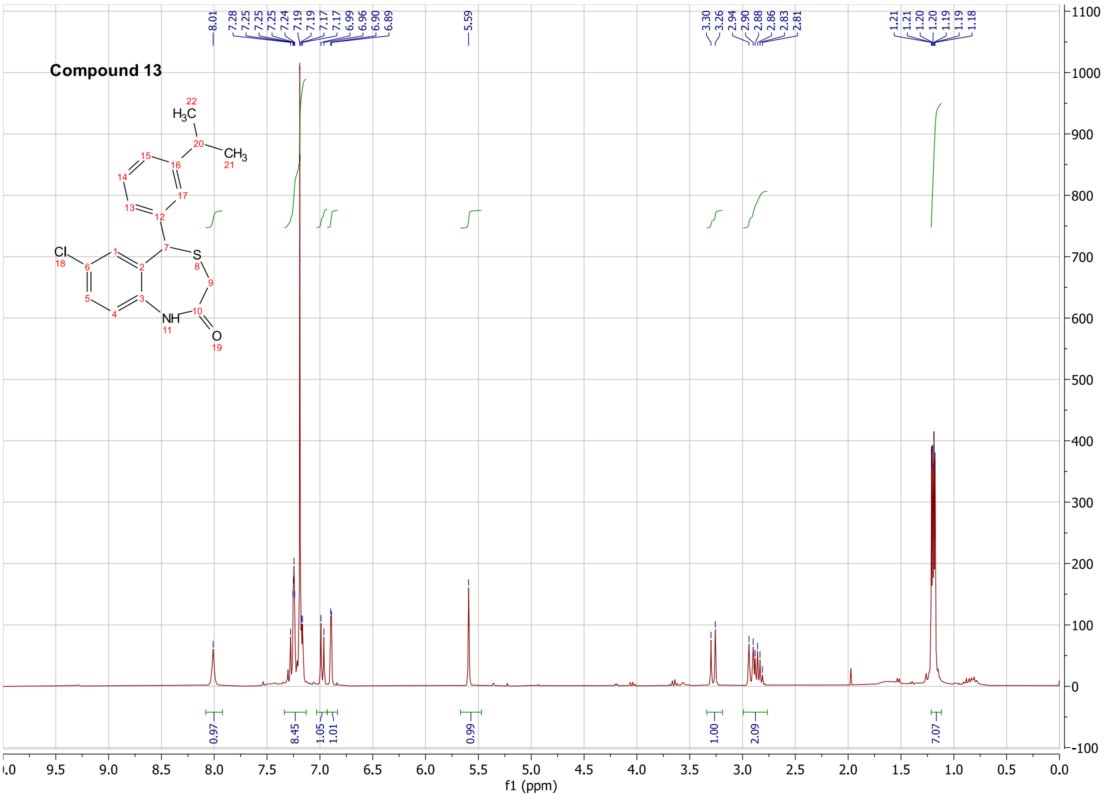
Compound 13
8.01
7.28
7.25
7.25
7.25
7.24
7.19
7.19
7.17
7.17
6.99
6.96
6.90
6.89
5.59
3.30
3.26
2.94
2.90
2.88
2.86
2.83
2.81
1.21
1.21
1.20
1.20
1.19
1.19
1.18
0.97
8.45
1.05
1.01
0.99
1.00
2.09
7.07
f1 (ppm)

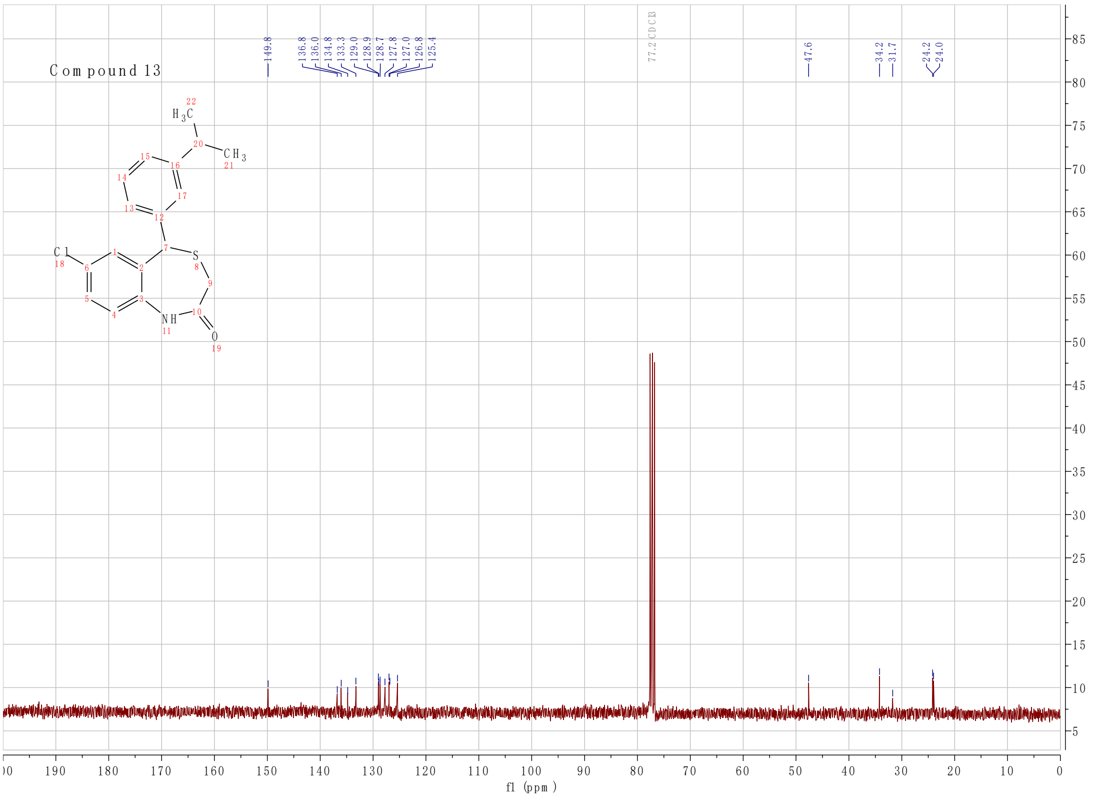
Compound 13
149.8
136.8
136.0
134.8
133.3
129.0
128.9
128.7
127.8
127.0
126.8
125.4
77.2 CDCl3
47.6
34.2
31.7
24.2
24.0
f1 (ppm)

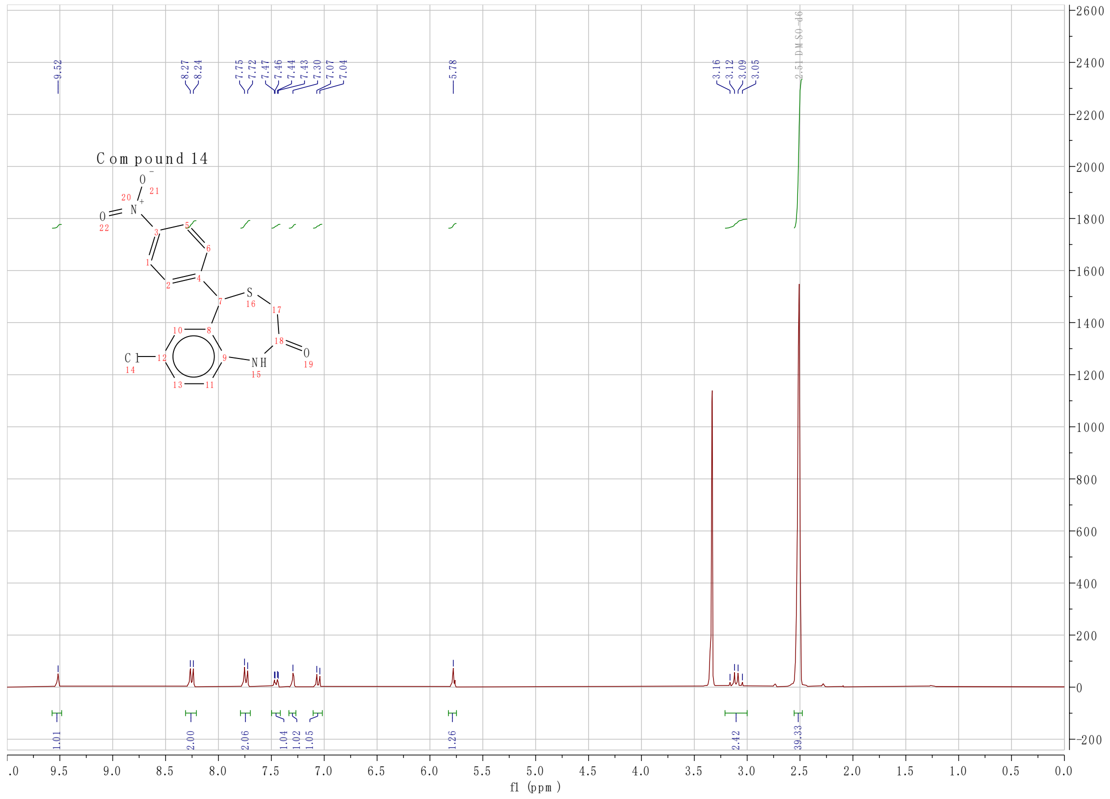

Compound 14
9.52
8.27
8.24
7.75
7.72
7.47
7.46
7.44
7.43
7.30
7.07
7.04
5.78
3.16
3.12
3.09
3.05
2.51 DMSO-d6
1.01
2.00
2.06
1.04
1.02
1.05
1.26
2.42
39.33
f1 (ppm)

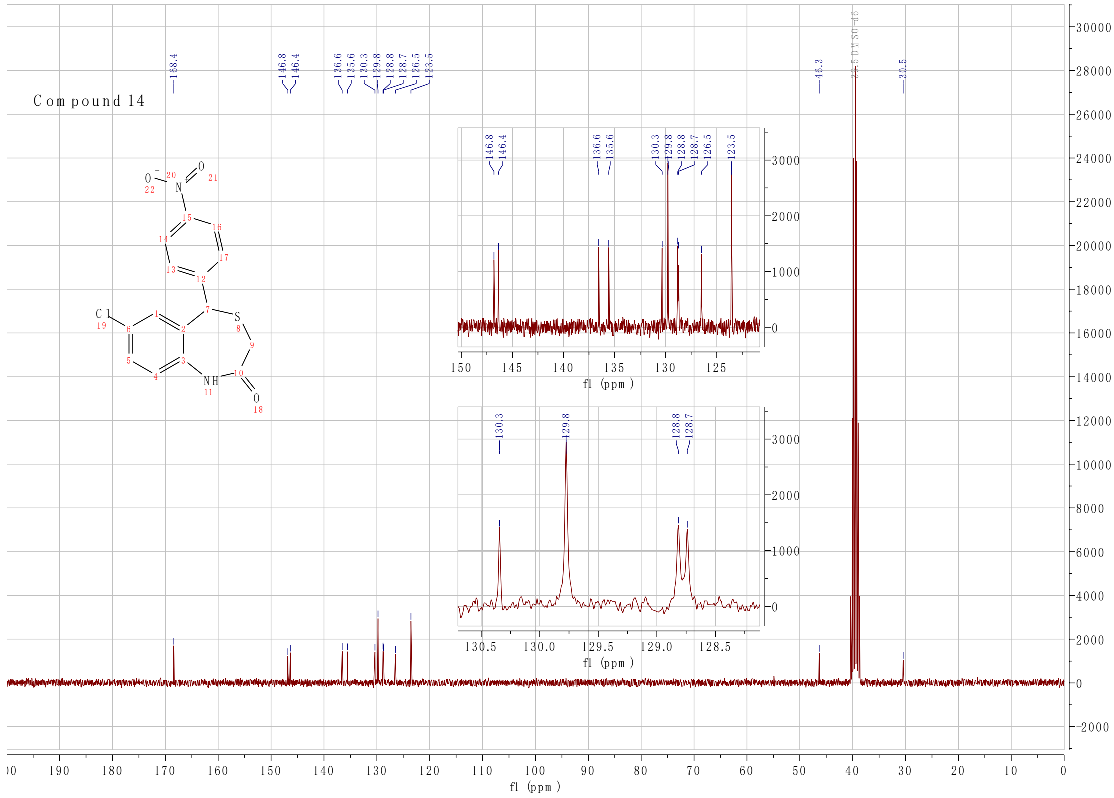

Compound 14
168.4
146.8
146.4
136.6
135.6
130.3
129.8
128.8
128.7
126.5
123.5
46.3
39.5 DMSO-d6
30.5
f1 (ppm)

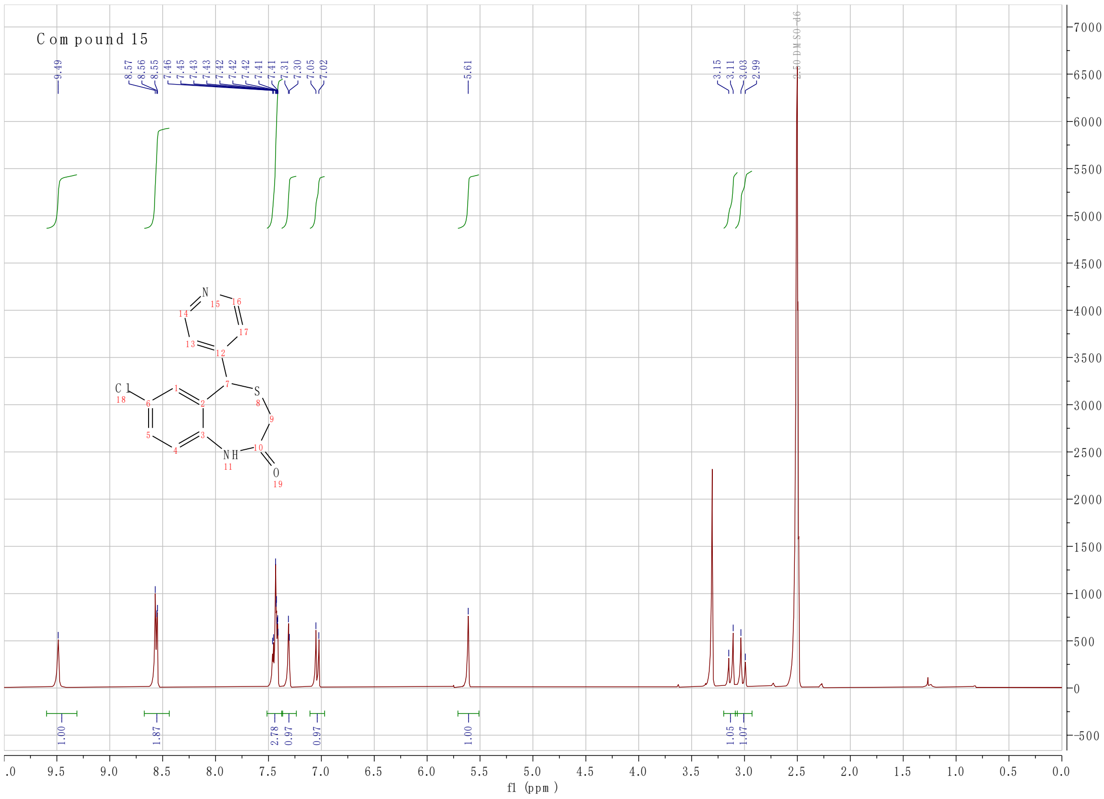
Compound 15
9.49
8.57 8.56 8.55 7.46 7.45 7.43 7.43 7.42 7.42 7.42 7.41 7.41 7.31 7.30 7.05 7.02
5.61
3.15 3.11 3.03 2.99
2.50 DMSO-d6
1.00 1.87 2.78 0.97 0.97 1.00 1.05 1.07
f1 (ppm)

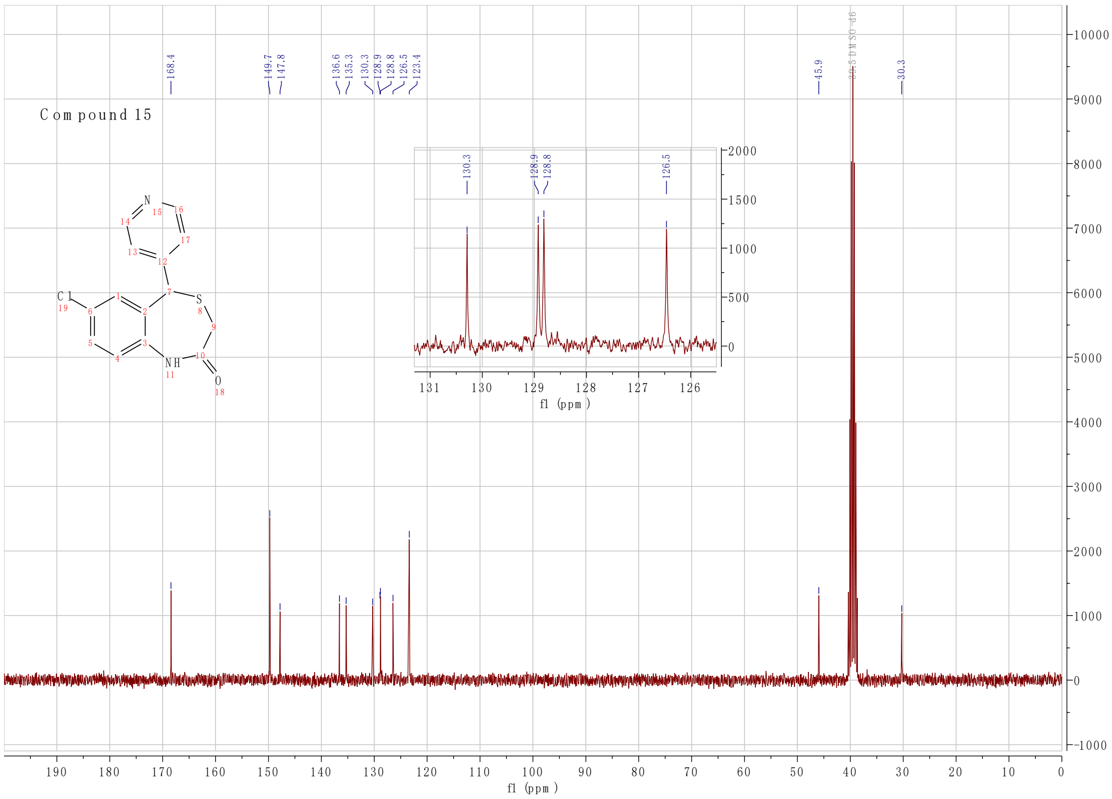
Compound 15
168.4
149.7 147.8
136.6 135.3 130.3 128.9 128.8 126.5 123.4
45.9
39.5 DMSO-d6
30.3
130.3 128.9 128.8 126.5
f1 (ppm)

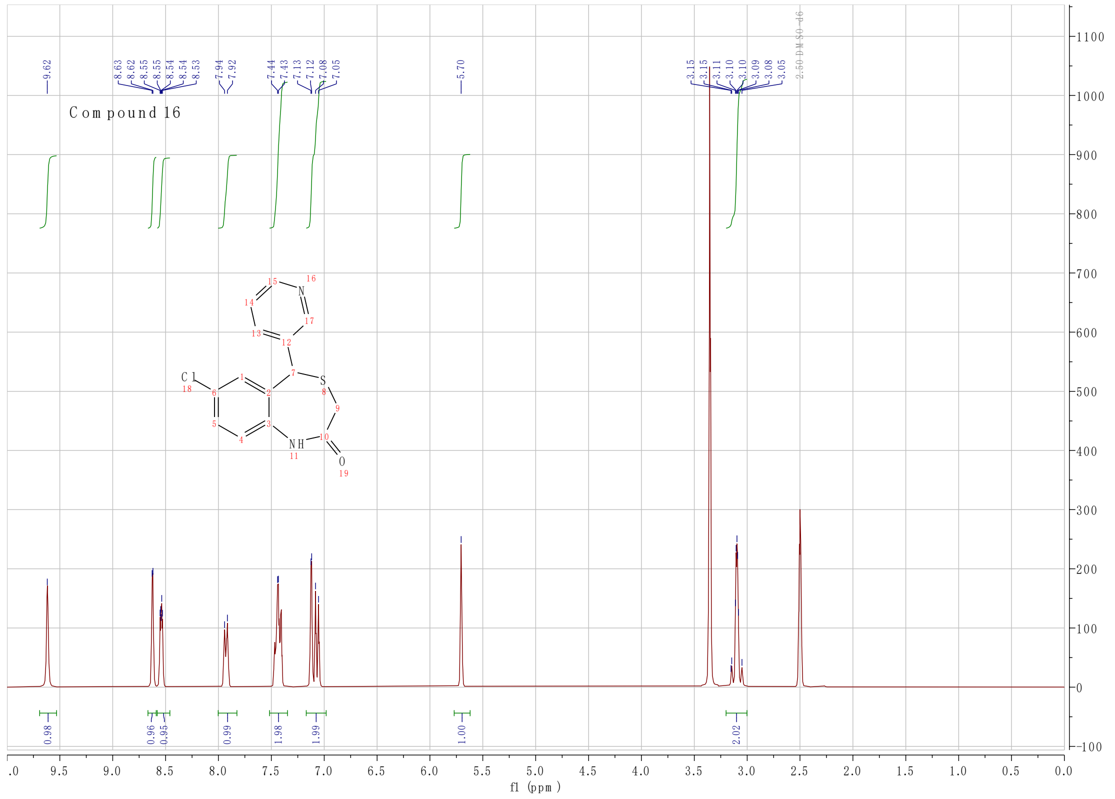
Compound 16
9.62
8.63
8.62
8.55
8.55
8.54
8.54
8.53
7.94
7.92
7.44
7.43
7.13
7.12
7.08
7.05
5.70
3.15
3.15
3.11
3.10
3.10
3.09
3.08
3.05
2.50 DMSO-d6
0.98
0.96
0.95
0.99
1.98
1.99
1.00
2.02
fl (ppm)

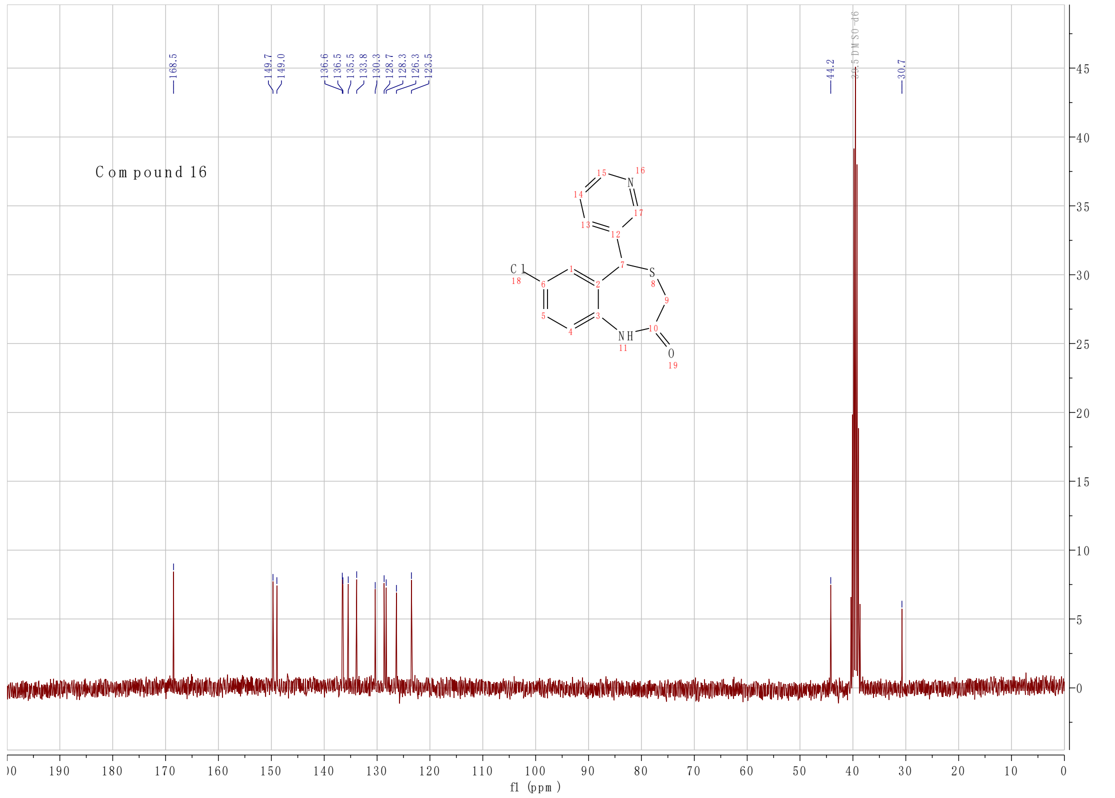
Compound 16
168.5
149.7
149.0
136.6
135.5
133.8
130.3
128.7
128.3
126.3
123.5
44.2
39.5 DMSO-d6
30.7
fl (ppm)

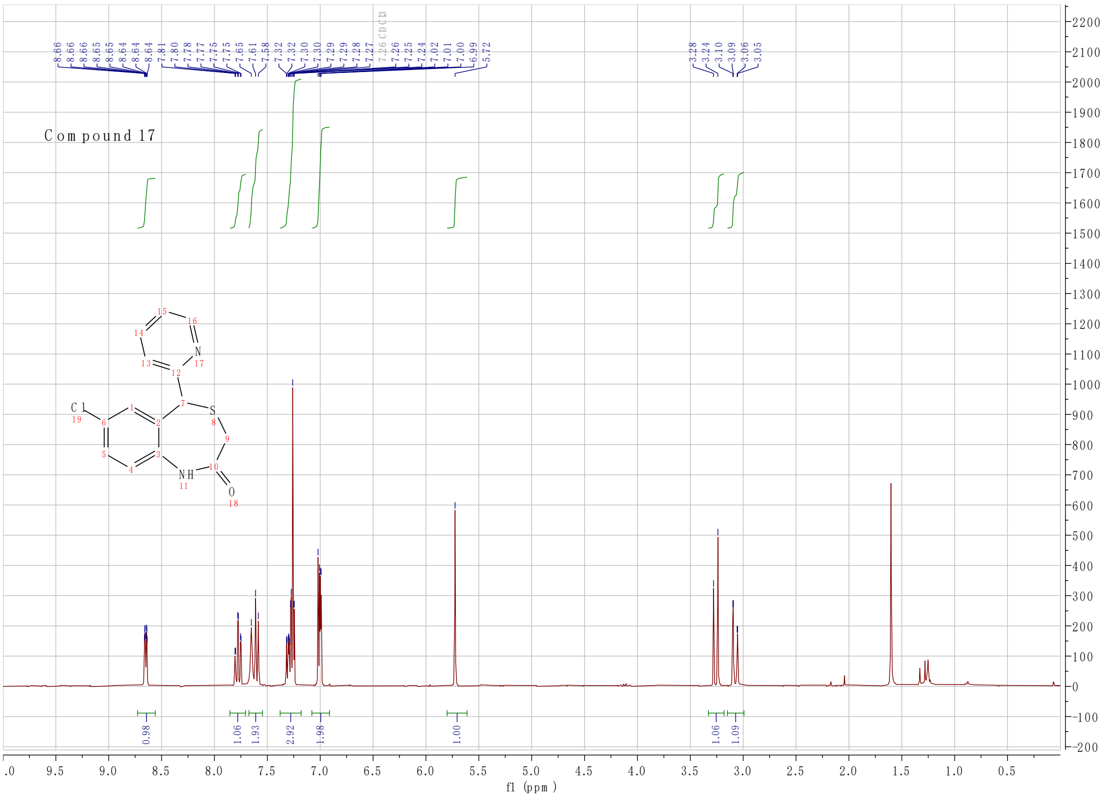

Compound 17
8.66 8.66 8.66 8.65 8.65 8.64 8.64 8.64 7.81 7.80 7.78 7.77 7.75 7.75 7.65 7.61 7.58 7.32 7.32 7.30 7.30 7.29 7.29 7.28 7.27 7.26 CDCl3 7.26 7.25 7.24 7.02 7.01 7.00 6.99 5.72
3.28 3.24 3.10 3.09 3.06 3.05
0.98 1.06 1.93 2.92 1.98 1.00 1.06 1.09
f1 (ppm)

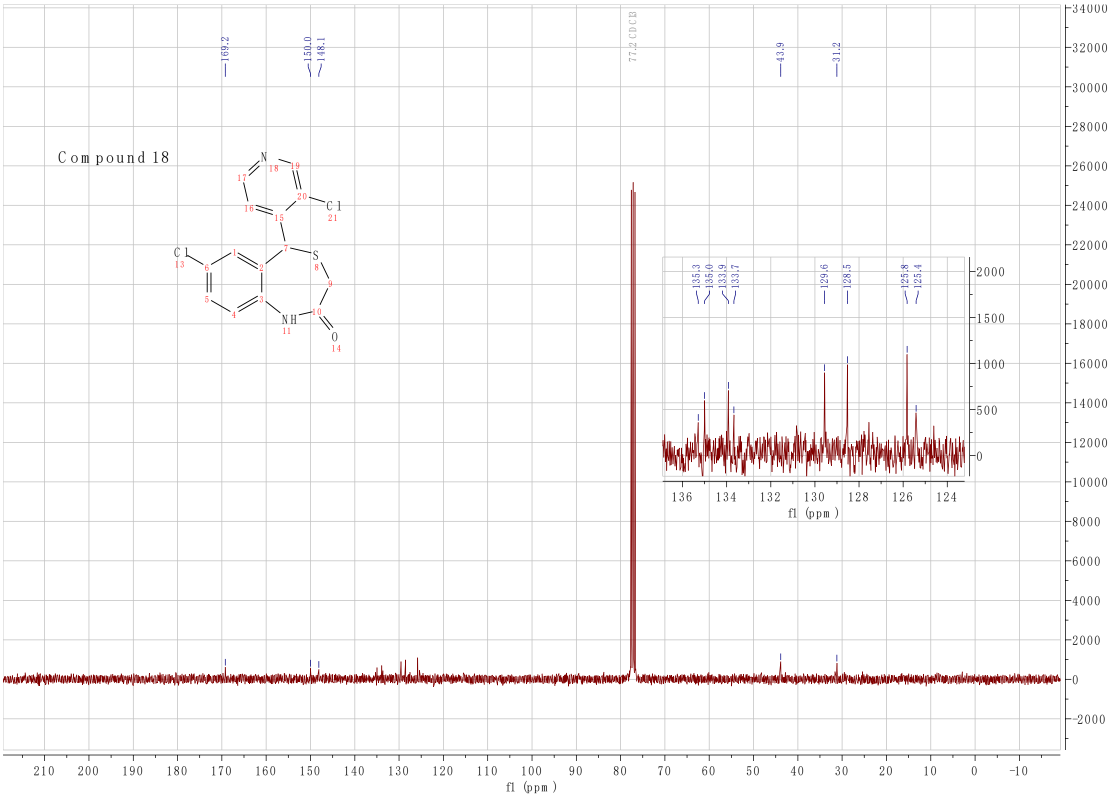

Compound 18
169.2 150.0 148.1 77.2 CDCl3 43.9 31.2
135.3 135.0 133.9 133.7 129.6 128.5 125.8 125.4
f1 (ppm)

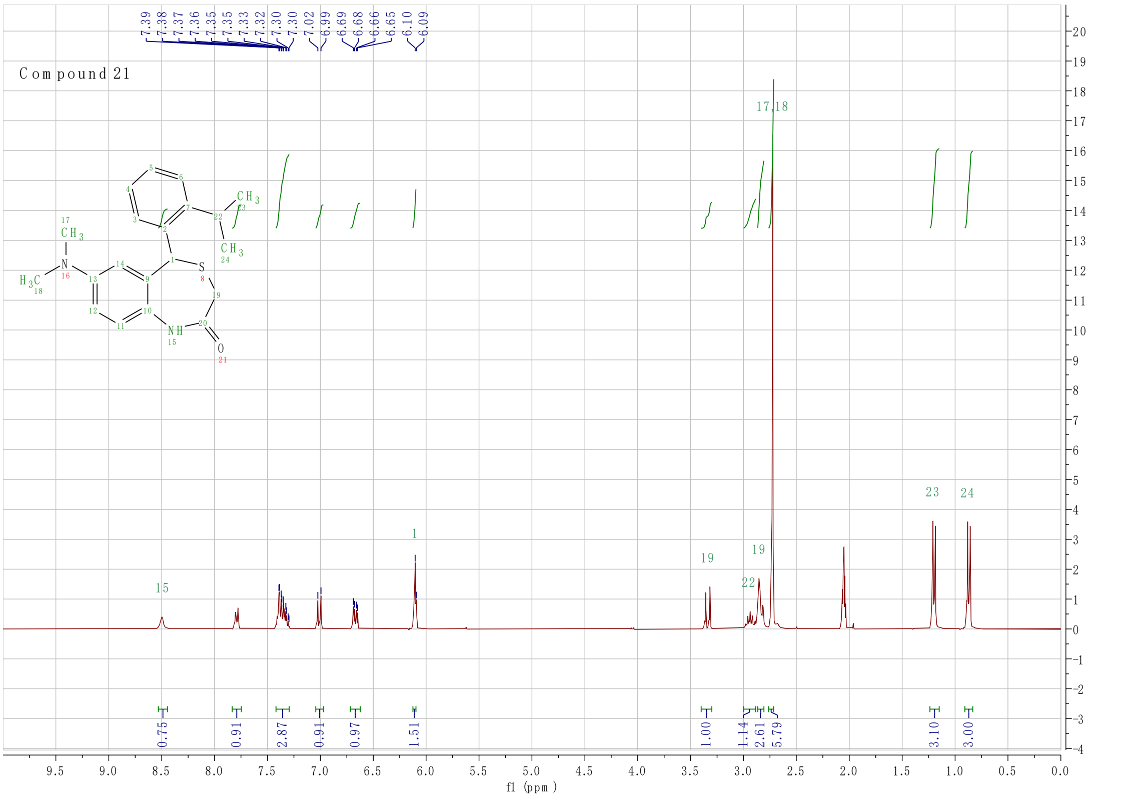
Compound 21
7.39 7.38 7.37 7.36 7.35 7.35 7.33 7.32 7.30 7.30 7.02 6.99 6.69 6.68 6.66 6.65 6.10 6.09
0.75 0.91 2.87 0.91 0.97 1.51 1.00 1.14 2.61 5.79 3.10 3.00
f1 (ppm)

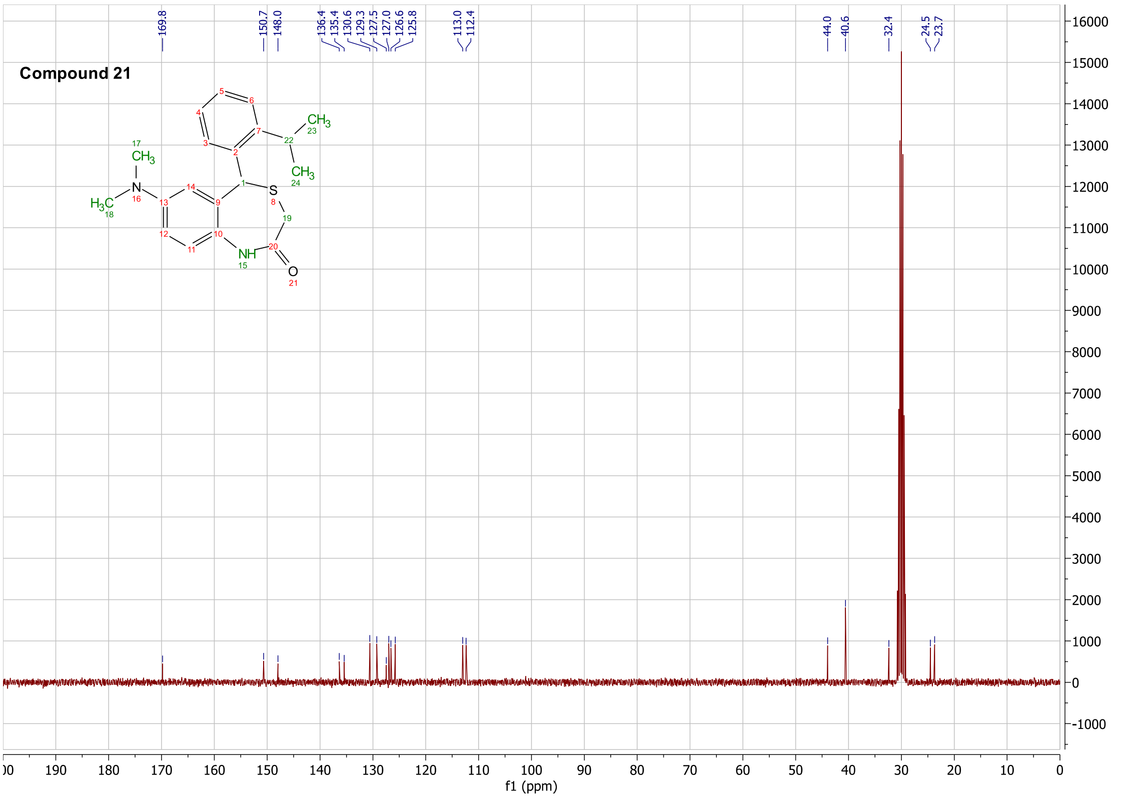
Compound 21
169.8 150.7 148.0 136.4 135.4 130.6 129.3 127.5 127.0 126.6 125.8 113.0 112.4 44.0 40.6 32.4 24.5 23.7
f1 (ppm)

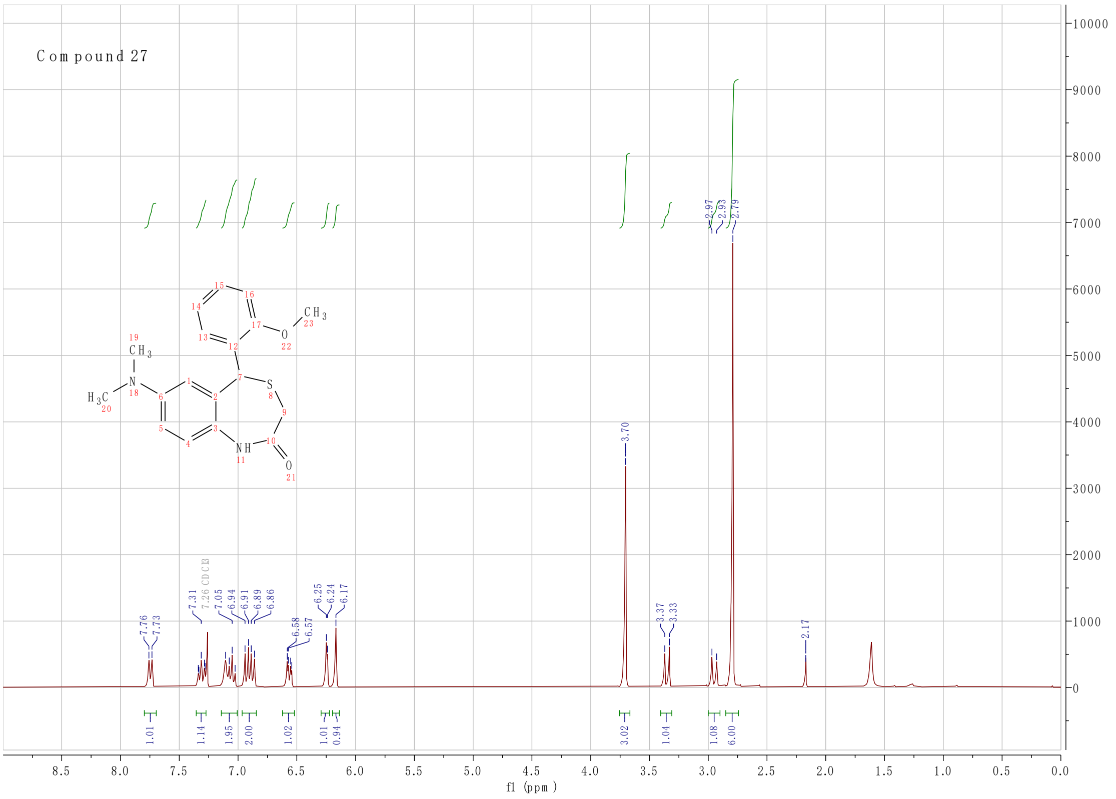
Compound 27
fl (ppm)

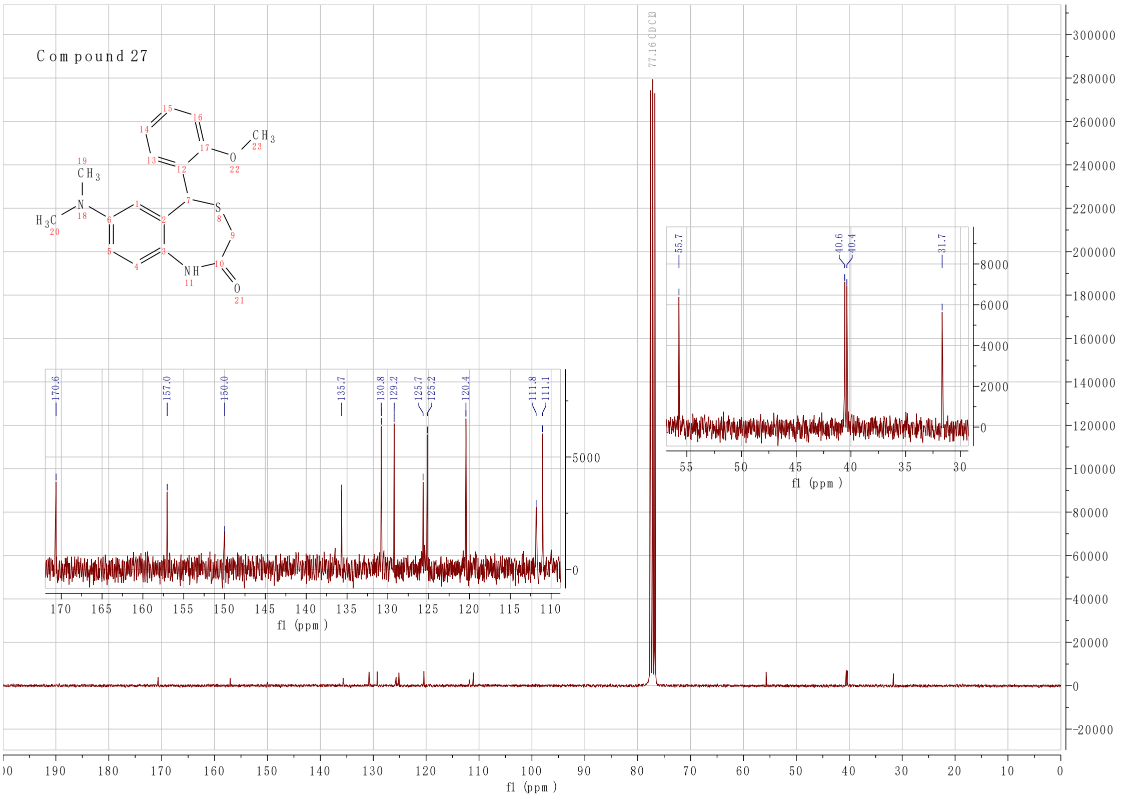
Compound 27
77.16 CDCl3
fl (ppm)

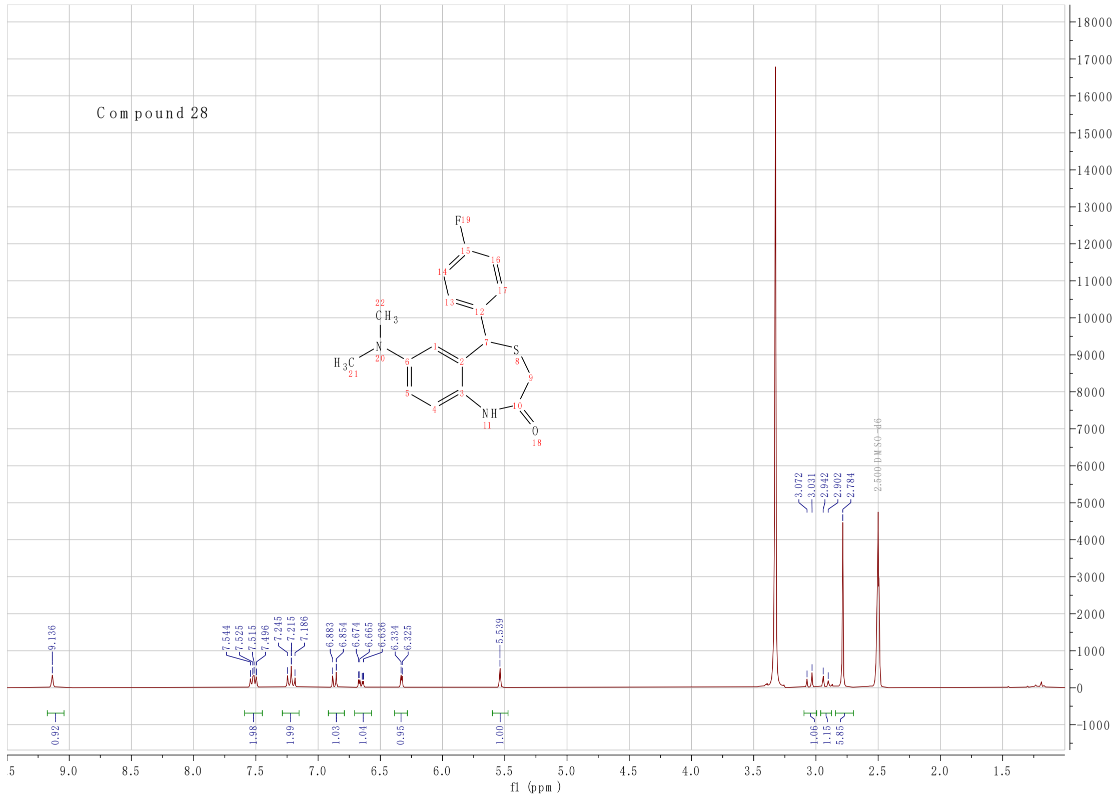
Compound 28
9.136
7.544 7.525 7.515 7.496 7.245 7.215 7.186
6.883 6.854 6.674 6.665 6.636 6.334 6.325
5.539
3.072 3.031 2.942 2.902 2.784
2.500 DMSO-d6
0.92 1.98 1.99 1.03 1.04 0.95 1.00 1.06 1.15 5.85
f1 (ppm)

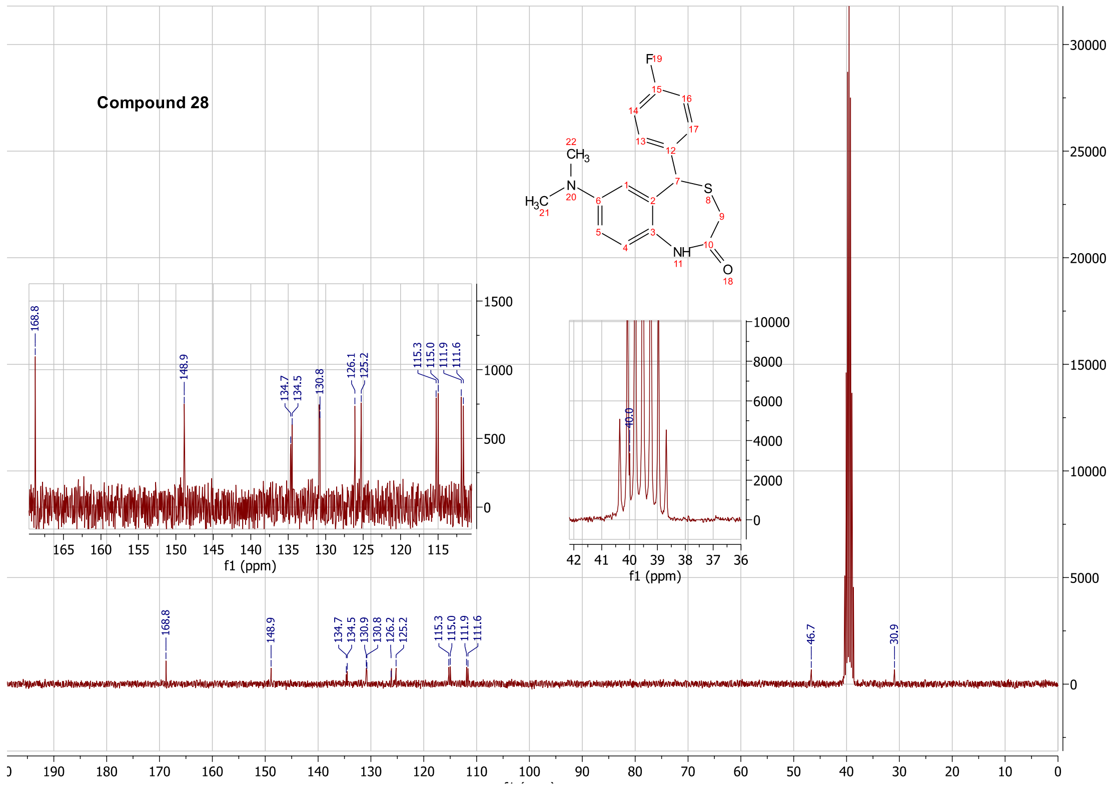
Compound 28
168.8
148.9
134.7 134.5 130.9 130.8 126.2 125.2
115.3 115.0 111.9 111.6
46.7
30.9
40.0
f1 (ppm)

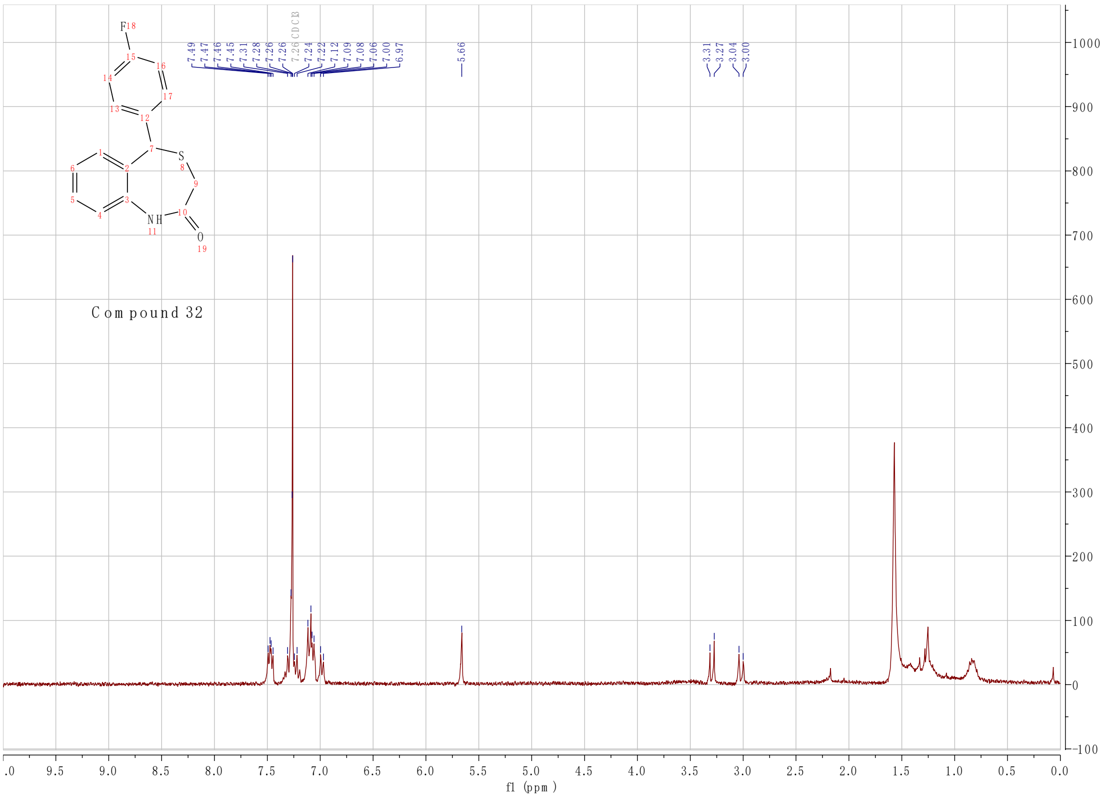
Compound 32
f1 (ppm)

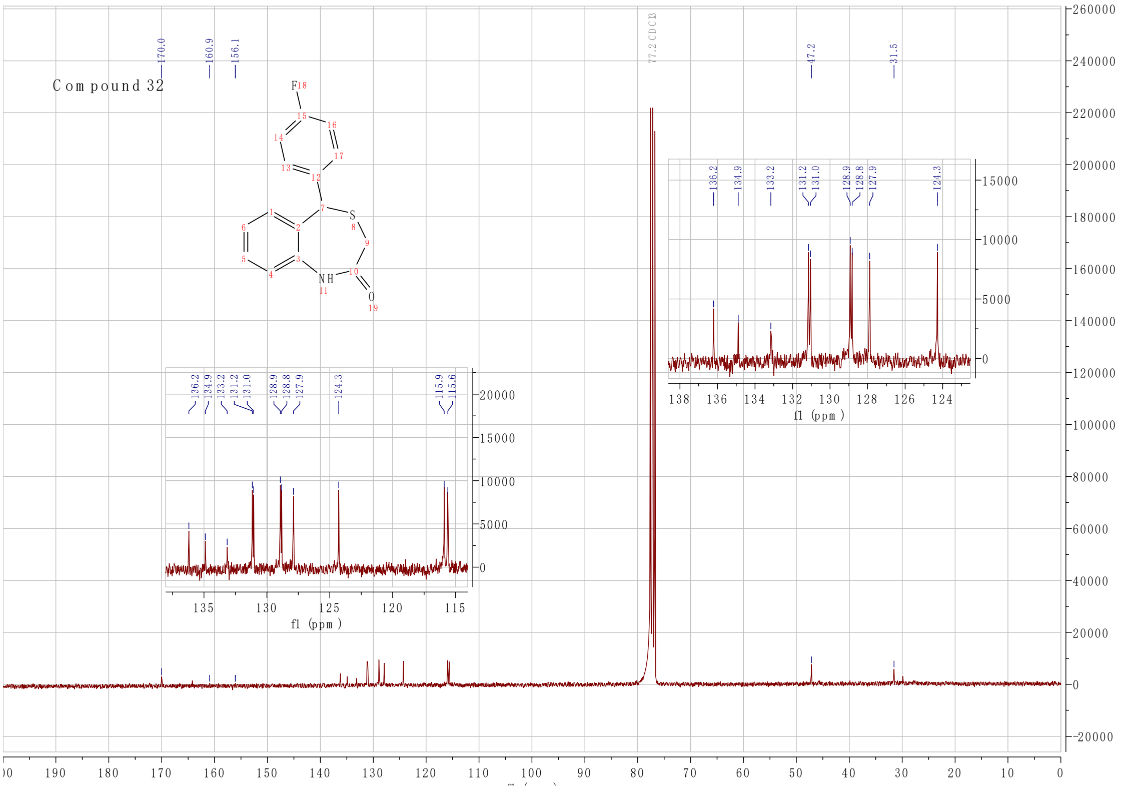
Compound 32
f1 (ppm)

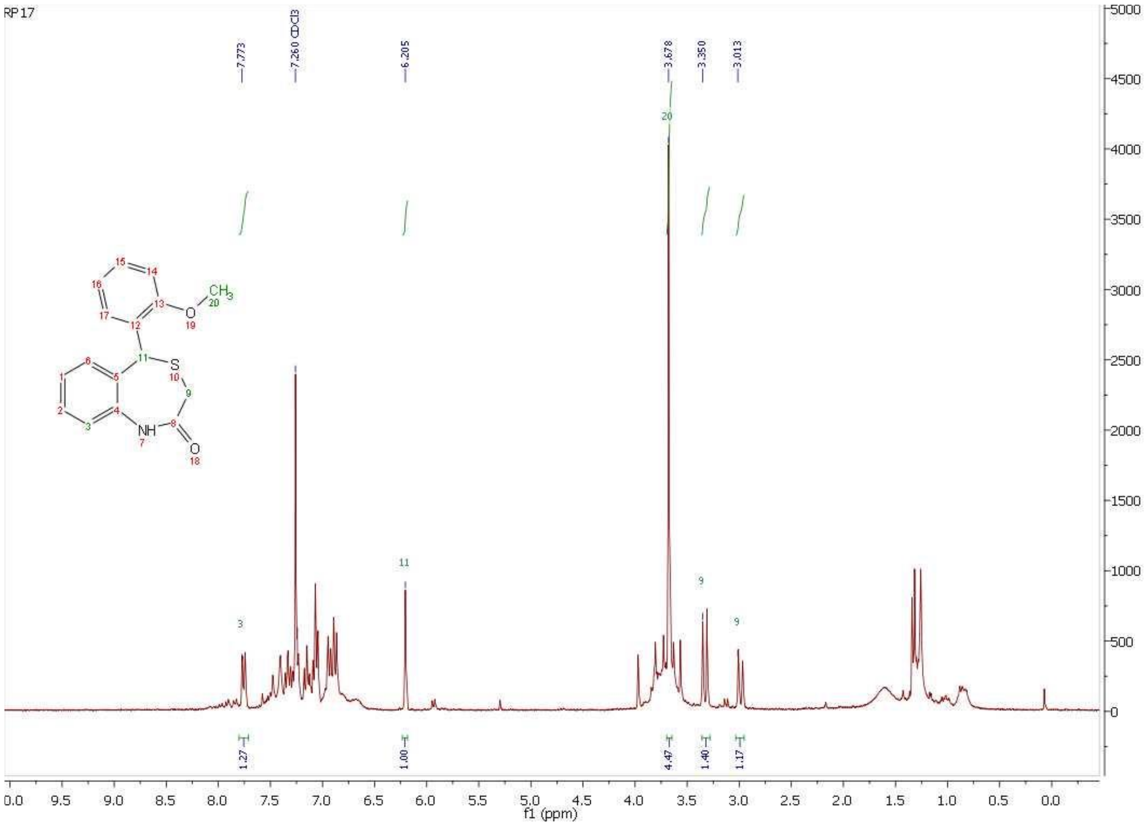
RP17
7.773
7.260 CDCl3
6.205
3.678
3.350
3.013
20
11
3
9
9
1.27
1.00
4.47
1.40
1.17
f1 (ppm)

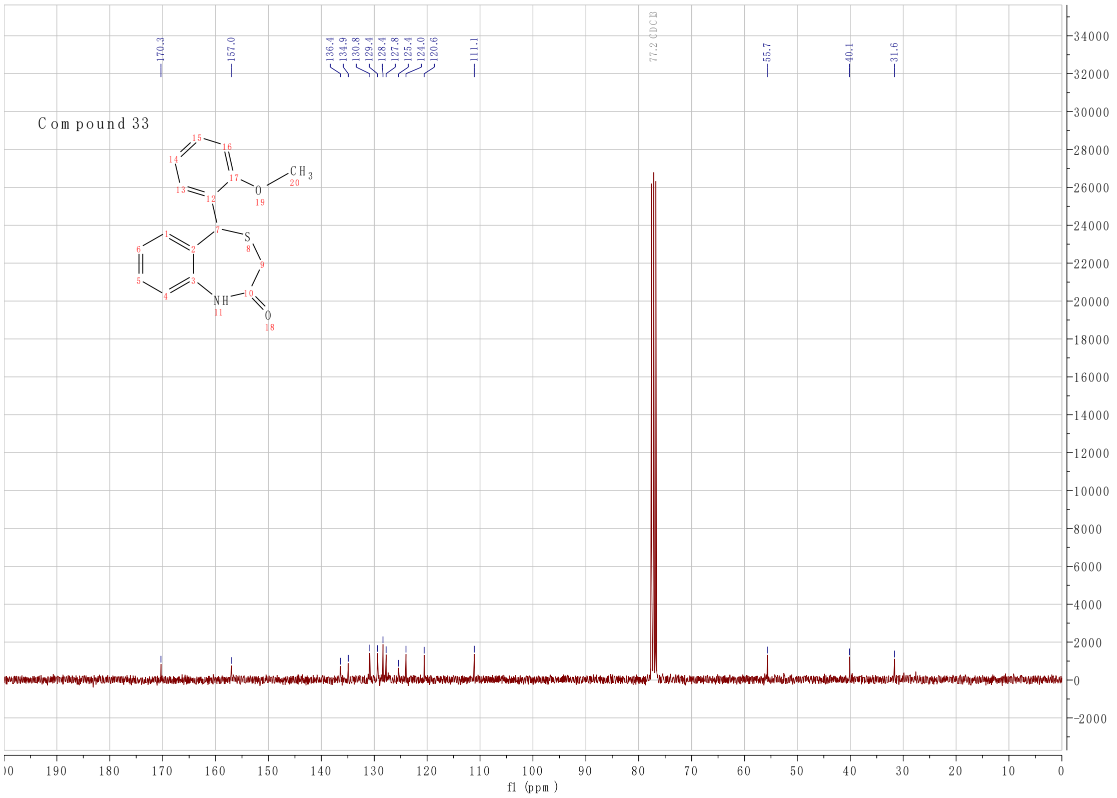
Compound 33
170.3
157.0
136.4
134.9
130.8
129.4
128.4
127.8
125.4
124.0
120.6
111.1
77.2 CDCl3
55.7
40.1
31.6
f1 (ppm)

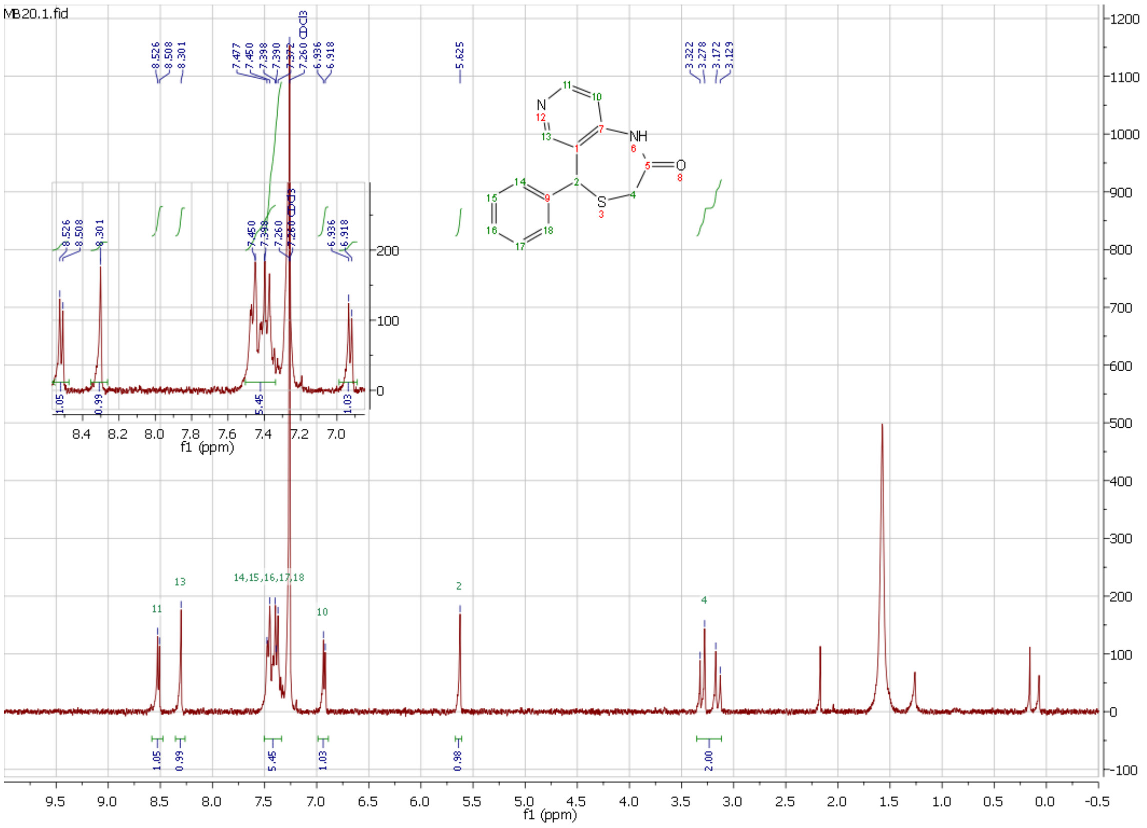
MB20.1.fid
8.526
8.508
8.301
7.477
7.450
7.398
7.390
7.372
7.260 CDCl3
6.936
6.918
5.625
3.322
3.278
3.172
3.129
11
13
14,15,16,17,18
10
2
4
1.05
0.99
5.45
1.03
0.98
2.00
f1 (ppm)

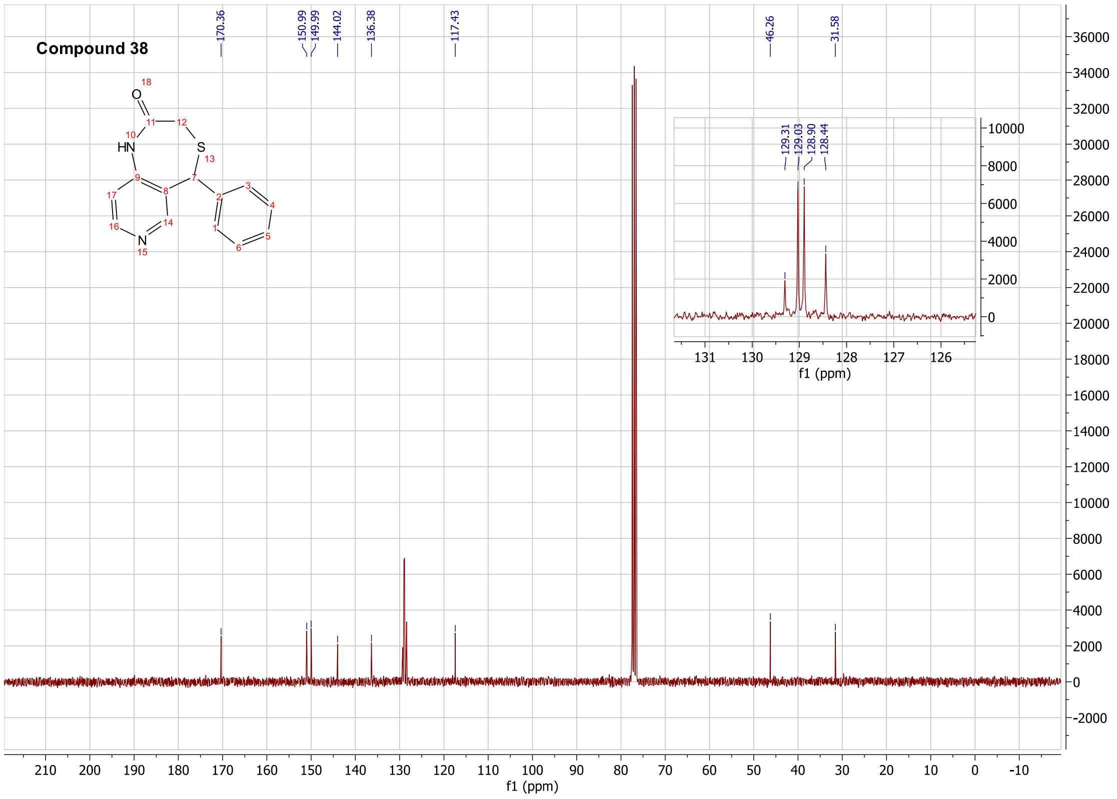
Compound 38
170.36
150.99
149.99
144.02
136.38
117.43
46.26
31.58
129.31
129.03
128.90
128.44
f1 (ppm)

Table S1. Prediction of log P and other Lipinski's parameters.

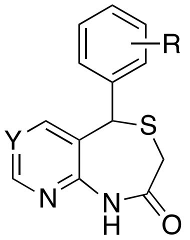

ITH12662

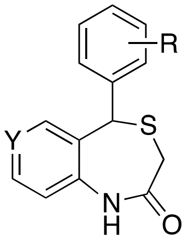

12–14, 21, 26–28, 32, 33, 38

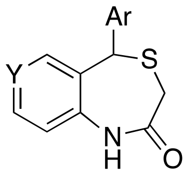

15–18

COMPOUNDS	Y	R	AR	DON.[A]	ACC.[B]	PSA (Å^2)[C]	CLOG P
**CGP37157**	C–Cl	2-Cl	-	1	2	54.4	4.36
**ITH12575**	C–Cl	2-^{i}Pr	-	1	2	54.4	5.00
**12**	C–Cl	4-^{i}Pr	-	1	2	54.4	5.00
**13**	C–Cl	3-^{i}Pr	-	1	2	54.4	5.00
**14**	C–Cl	4-NO_2	-	1	4	100.2	3.70
**21**	C–NMe_2	2-^{i}Pr	-	1	3	57.6	4.51
**26**	C–NMe_2	2-Cl	-	1	3	57.6	3.86
**27**	C–NMe_2	2-OMe	-	1	4	66.9	3.10
**28**	C–NMe_2	4-F	-	1	3	57.6	3.40
**32**	C–H	4-F	-	1	2	54.4	3.30
**33**	C–H	2-OMe	-	1	3	63.6	2.99
**38**	N	H	-	1	3	67.3	1.93
**15**	C–Cl	H	4-y	1	3	67.3	2.54
**16**	C–Cl	H	3-y	1	3	67.3	2.54
**17**	C–Cl	H	2-y	1	3	67.3	2.67
**18**	C–Cl	2-Cl	4-y	1	3	67.3	3.14
**ITH12662**	C–Cl	4-F	-	1	3	67.3	3.28

[a] Number of H-bonds-donating groups; [b] Number of H-bonds-accepting groups; [c] Polar surface Area (according to the Hopkins-modified Lipinski's rules of five, PSA of drug-like compounds should be below 150).

References and notes

[1] Han, C.; Buchwald, S.L. Negishi Coupling of Secondary Alkylzinc Halides with Aryl Bromides and Chlorides. J Am Chem Soc **2009***, 131*, 7532-7533.

[2] Ma, F.; Xie, X.; Zhang, L.; Peng, Z.; Ding, L.; Fu, L.; Zhang, Z. Palladium-Catalyzed Amidation of Aryl Halides using 2-Dialkylphosphino-2'-Alkoxyl-1,1'-Binaphthyl as Ligands. J Org Chem **2012***, 77*, 5279-5285.

[3] Gowda, B.T.; Usha, K.M.; Jayalakshmi, K.L. H and 13 C NMR Spectral Studies on N-(Aryl)-Substituted Acetamides, C 6 H 5 NHCOCH 3-i X i and 2/4-XC 6 H 4 NHCOCH 3-i X i (Where X = Cl Or CH 3 and i = 0, 1, 2 Or 3). Z. Naturforsch **2003***, 58*, 801.

[4] Turner, J.A. Regiospecific Electrophilic Substitution of Aminopyridines: Ortho Lithiation of 2-, 3-, and 4-(Pivaloylamino)Pyridines. J. Org. Chem. **1983***, 48*, 3401-3408.
